# Reaction Time Variability and Electrophysiological Indices of Performance Monitoring in Youths With Attention‐Deficit/Hyperactivity Disorder and Obsessive‐Compulsive Disorder

**DOI:** 10.1111/psyp.70403

**Published:** 2026-09-24

**Authors:** Gregory L. Hanna, Yanni Liu, Barbara S. Hanna, Paul D. Arnold, William J. Gehring

**Affiliations:** ^1^ Department of Psychiatry University of Michigan Ann Arbor Michigan USA; ^2^ Mathison Centre for Mental Health Research and Education University of Calgary Calgary Alberta Canada; ^3^ Department of Psychology University of Michigan Ann Arbor Michigan USA

**Keywords:** attention‐deficit/hyperactivity disorder, EEG, error positivity, error‐related negativity, obsessive‐compulsive disorder, reaction time variability

## Abstract

Attention‐deficit/hyperactivity disorder (ADHD) and obsessive‐compulsive disorder (OCD) are common psychiatric disorders that may be differentiated by neurocognitive measures and electrocortical indices of performance monitoring. Increased reaction time variability (RTV) is a consistent finding in studies of ADHD. The error‐related negativity (ERN) and error positivity (Pe) are components of the event‐related potential (ERP) following an error that are potential mechanistic biomarkers for both disorders. The correct response negativity (CRN) and correct positivity (Pc) are components of the ERP following a correct response that are less well understood. The study examined RTV, accuracy, and neural indices of error monitoring using an Eriksen flanker task in 84 ADHD cases, 90 OCD cases, and 136 matched healthy controls (HC) ages 8 to 18 years. Both correct and error trial RTV were significantly increased in ADHD cases compared to HC, whereas flanker task accuracy was significantly decreased in OCD cases relative to HC. Compared to HC, CRN amplitude was significantly increased, and Pe amplitude was significantly decreased in ADHD cases. ERN amplitude was significantly increased in OCD cases compared to HC. Compared to HC, the difference between Pe and Pc amplitudes was significantly decreased in both ADHD and OCD cases, which may reflect defects in the post‐decisional evidence accumulation process that impair decision accuracy or subsequent behavioral adjustments in those disorders. In two multiple linear regression analyses, correct trial CVRT had significant associations with age, ADHD behaviors, and CRN, Pe, and Pc amplitudes. The study provides new evidence of increased RTV in ADHD, differential alterations in error‐related and correct‐related brain activity in ADHD and OCD, and a robust association between RTV and the error monitoring neural system.

## Impact Statement

Attention‐deficit/hyperactivity disorder (ADHD) and obsessive‐compulsive disorder (OCD) may be differentiated by neurocognitive measures and neural indices of performance monitoring. The study provides evidence of increased reaction time variability in ADHD, decreased flanker task accuracy in OCD, and differential alterations in error‐related and correct‐related brain activity in the two disorders. It also describes a novel association between reaction time variability and the error monitoring neural system.

## 1 Introduction

Attention‐deficit/hyperactivity disorder (ADHD) is a heterogeneous externalizing disorder that is diagnosed in about 11% of U.S. children and adolescents and is comorbid with at least one other psychiatric disorder in about 78% of cases (Danielson et al. 2024). ADHD is characterized by inattentive, impulsive, and hyperactive behaviors that begin before age 12 years and vary in their severity and persistence between individuals; however, intraindividual behavioral variability or inconsistency may be a core and stable feature of the disorder (Kofler et al. 2013; Tamm et al. 2025). Obsessive‐compulsive disorder (OCD) is a heterogeneous internalizing disorder with lifetime prevalence rates ranging from 1% to 3% (Ruscio et al. 2010) and about 25% of cases starting by age 14 years (Kessler et al. 2005). OCD involves recurrent intrusive thoughts and repetitive behaviors or mental acts that vary in their content and are comorbid with at least one other psychiatric disorder in about 69% of cases (Sharma et al. 2021).

An early model placed ADHD and OCD at opposing ends of a hypothesized impulsivity‐compulsivity spectrum based on neurocognitive measures (Robbins et al. 2012). Although neuroimaging studies have compared the two disorders in pediatric samples, similar studies have not been done to our knowledge comparing youths with ADHD and OCD using standard measures of intrasubject reaction time variability (RTV) and neural markers of performance monitoring (Norman et al. 2016). The study reported herein builds upon our previous studies of neural indices of error monitoring in youths with ADHD (Liu et al. 2020) and OCD (Hanna et al. 2026). Research on such mechanistic biomarkers in ADHD and OCD may clarify their pathogenesis, improve diagnostic and preventive strategies, and identify treatment targets (Clayson 2025; Pine and Leibenluft 2015).

### 1.1 Reaction Time Variability in ADHD

Increased RTV is one of the most consistent findings in studies of children and adults with ADHD (Kofler et al. 2013; Tamm et al. 2012). It is considered a marker of attentional fluctuations or lapses that may reflect impaired top‐down attentional control (Tamm et al. 2025) and discriminate patients with ADHD from controls better than other neuropsychological measures (Pievsky and McGrath 2018). Higher RTV is associated with multiple negative sequelae of ADHD, including impaired social interactions (Munch et al. 2025; Tamm et al. 2019), impaired reading decoding (Tamm et al. 2014), academic underachievement (Sjöwall et al. 2017), and poorer overall functioning (van Lieshout et al. 2017). A twin study provided an RTV heritability estimate of 50% (Kuntsi et al. 2006); a recent genome‐wide association study identified seven significant loci for RTV (Wootton et al. 2023). Furthermore, RTV was found to mediate 60% of the relationship between ADHD polygenic risk scores and ADHD traits, providing support for considering RTV as an endophenotype for ADHD (Moses et al. 2022).

### 1.2 Neurophysiological Correlates of Performance Monitoring

Performance monitoring in the context of cognitive control denotes neural processes that support the continuous monitoring of thoughts and actions (Bellato et al. 2021; Gehring et al. 2012). Two components of the event‐related potential (ERP) involved in performance monitoring are the error‐related negativity (ERN or Ne), a sharp negative deflection in the response‐locked ERP waveform that peaks within 100 ms after error commission and is maximal at frontocentral electrodes, and the error positivity (Pe), a larger positive deflection in the response‐locked ERP waveform that peaks between 200 and 500 ms after an erroneous response and is maximal at centroparietal electrodes (Desender et al. 2021; Falkenstein et al. 1991; Gehring et al. 1993, 2012). The correct response negativity (CRN) and correct positivity (Pc) that occur after a correct response in the same time windows as the ERN and Pe, respectively, are distinguished from their counterparts by having lower amplitudes (Desender et al. 2021; Gehring et al. 2012). A genetic analysis found substantial heritability in the ERN, CRN, and Pe ranging from 40% to 60%, suggesting those ERP components may serve as endophenotypes in genetic studies of childhood psychopathology (Anokhin et al. 2008).

The ERN has been described as a neural marker of error monitoring processes (Falkenstein et al. 1991; Gehring et al. 1993), reinforcement learning (Holroyd and Coles 2002), error‐related distress (Bartholow et al. 2005), and the motivational significance of errors (Hajcak et al. 2005). The ERN becomes more negative and the CRN less negative throughout childhood and adolescence, indicating a prolonged maturation of that part of the system underlying error monitoring (Boen et al. 2022; Overbye et al. 2019; Tamnes et al. 2013). The ERN is not correlated in general with reaction time or RTV; however, some studies have found a higher CRN amplitude is associated with slower responses (Files et al. 2021; Luu et al. 2000). Studies in humans and macaques have provided evidence that error neurons in the medial frontal cortex, specifically in the pre‐supplementary motor area and dorsal anterior cingulate cortex (ACC), constitute single‐neuron correlates of the ERN with spiking activity of error neurons predicting the scalp‐measured ERN in both species (Fu et al. 2019, 2023).

The Pe is posited to reflect the post‐decisional evidence accumulation process that is sensitive to decision accuracy, decision confidence, and subsequent adaptation in behavior (Desender et al. 2021; Steinhauser and Yeung 2010). It has a robust association with error detection that varies with the level of confidence that an error has been made, with a higher Pe amplitude reflecting greater certainty about a mistake (Boldt and Yeung 2015; Murphy et al. 2012). In contrast to the ERN, the Pe does not increase in magnitude in older children or adolescents, indicating differential maturation of those ERP components (Boen et al. 2022). A source‐localization study suggested the Pe is generated primarily in the rostral ACC (Herrmann et al. 2004). Recent studies found that the Pe and error awareness can occur in the absence of an ERN, providing evidence of two independent systems of error monitoring (Di Gregorio et al. 2018; Dumsky et al. 2025). Another recent study found the CRN and Pc are more strongly correlated than the ERN and Pe (LoTemplio et al. 2026). The results altogether suggest all four ERP components should be considered separately in studies of error monitoring and RTV.

### 1.3 Electrocortical Indices of Performance Monitoring in ADHD and OCD

Reduced ERN and Pe amplitudes have been found in studies of children and adults with ADHD using choice reaction time tasks, suggesting the ERN and Pe may serve as biomarkers for the disorder (Bellato et al. 2021; Figuracion et al. 2024; Geburek et al. 2013; Kaiser et al. 2020; Liu et al. 2020; Lutz et al. 2021). However, one meta‐analysis noted a blunted Pe was associated more consistently with ADHD than a blunted ERN (Kaiser et al. 2020). We also found in our study of ADHD an increased CRN amplitude, average ERN amplitude, and decreased ERN minus CRN (ΔERN), as well as a decreased Pe amplitude and decreased Pe minus Pc (ΔPe) (Liu et al. 2020).

Increased ERN amplitudes have been found in studies of children and adults with OCD using choice reaction time tasks eliciting response conflict, indicating the ERN may serve as a biomarker for OCD (Bellato et al. 2021; Hajcak et al. 2008; Hanna et al. 2026; Lamothe et al. 2025; Michael et al. 2021; Riesel 2019). Studies of adults with OCD have noted either normal or increased accuracy on response conflict tasks relative to HC (Bellato et al. 2021; Riesel 2019). Studies of older children and adolescents with OCD, however, have shown decreased accuracy compared to HC (Hanna et al. 2026; Lamothe et al. 2025). An increased Pe amplitude was reported in a meta‐analysis of seven studies of adults and children with OCD or obsessive‐compulsive symptoms using choice reaction time tasks eliciting response conflict (Bellato et al. 2021). In contrast, we found in our study a decreased ΔPe in youths with OCD compared to HC that was associated with lower flanker task accuracy (Hanna et al. 2026).

### 1.4 Study Aims and Hypotheses

Numerous studies have found that increased RTV is an important mechanistic endophenotype in ADHD that may reflect impaired top‐down attentional control (Kofler et al. 2013; Moses et al. 2022; Tamm et al. 2025). Furthermore, it may be biologically parsimonious for the neural system underlying error detection and compensation to be associated with RTV. If that is the case, the CRN may be more sensitive than the ERN to delayed responses on correct trials that reflect the attentional lapses associated with ADHD (Kofler et al. 2013; Tamm et al. 2012). Hence, this study was done with 84 youths with a lifetime diagnosis of ADHD, 90 youths with a lifetime diagnosis of OCD, and 136 HC using an Eriksen flanker task with substantial group differences in RTV, accuracy, and neural indices of error monitoring (Hanna et al. 2026; Liu et al. 2020). The coefficient of variation of reaction time (CVRT) was calculated as the standard deviation of reaction time divided by mean reaction time on correct trials and was used as the primary index of intrasubject RTV as it controls for differences in mean reaction time (Elmaghrabi et al. 2020).

The first aim was to compare flanker task accuracy and CVRT in the three groups. It was predicted that compared to HC, accuracy would be decreased in OCD cases and correct and error trial CVRT would be increased in ADHD cases. The second aim was to compare the four neural indices of error monitoring (ERN, CRN, Pe, and Pc amplitudes) in the three groups. It was predicted that compared to HC, CRN amplitude would be increased (more negative) in ADHD cases, ERN amplitude would be increased (more negative) in OCD cases, Pe amplitude would be decreased (less positive) in ADHD cases, and the ΔPe would be decreased in both ADHD and OCD cases. The third aim was to do a multiple linear regression analysis with all participants to examine the association of flanker task correct trial CVRT with age, psychiatric diagnosis, and the four brain potentials. It was predicted that correct trial CVRT would have significant associations with age, lifetime ADHD diagnosis, and CRN and Pe amplitudes. The fourth aim was to do a similar multiple linear regression analysis to examine the association of correct trial CVRT with age, the four brain potentials, and CBCL/6–18 *DSM*‐Oriented Scale scores to assess the specificity of the relationship between correct trial CVRT and Attention Deficit/Hyperactivity (AD/H) Problems scores (Achenbach and Rescorla 2001). It was predicted that correct trial CVRT would have significant associations with age, AD/H Problems scores, and CRN and Pe amplitudes.

## 2 Method

### 2.1 Participants

Patients with either ADHD or OCD were recruited from the Department of Psychiatry at the University of Michigan and surrounding community. HC were recruited from the surrounding community and were matched to patients by age. Participants were recruited using flyers and UM Health Research Studies (http://www.UMHealthResearch.org). Participants or their parents gave written informed consent in accordance with the Declaration of Helsinki. All tasks and procedures were approved by the University of Michigan Medical School Institutional Review Board. Participants were paid for their interviews and electrophysiological recordings. Participants were excluded if they made fewer than ten errors or had less than 60% accuracy on the flanker task. The final sample consisted of 153 males and 157 females of age 8–18 years, with an ethnic/racial breakdown that was 87% Caucasian, 1% African American, 4% Latino, 1% Asian, and 7% multiracial. Female participants were significantly older than male participants (*t*(308) = 3.71, *p* = 0.0002, Cohen's *d* = 0.36). All participants lived with at least one English‐speaking biological parent willing to participate in the research. Tables 1, 2, 3 summarize the demographic, clinical, behavioral, and ERP data for the participants.

**TABLE 1:** Demographic and clinical data in participants with ADHD, participants with OCD, and HC participants.

Variable	ADHD	OCD	HC	ADHD vs. OCD vs. HC	
	(*n* = 84)	(*n* = 90)	(*n* = 136)	Participants	*p*
	Mean (SD) or %	Mean (SD) or %	Mean (SD) or %	Test statistic	
*Demographic and age at onset data*					
Sex, % male	64.3%	35.6%	49.3%	*χ* ^2^_2_ = 14.6	0.0007
Age, years	13.4 (3.0)	13.8 (2.9)	13.5 (2.9)	*F*_2,307_ = 0.37	0.69
Age at onset of ADHD symptoms, years	5.6 (1.8)				
Age at onset of OCD symptoms, years		8.0 (3.2)			
*Child behavior checklist data*					
Total score	50.8 (27.3)****, ^††††^	34.9 (21.3)****	9.6 (9.5)	*F*_2,305_ = 125.5	< 0.0001
Internalizing score	13.1 (9.6)****	14.5 (9.3)****	3.0 (3.1)	*F*_2,305_ = 83.5	< 0.0001
Externalizing score	13.9 (10.4)****, ^††††^	6.4 (6.3)****	2.6 (3.6)	*F*_2,305_ = 71.9	< 0.0001
Obsessive‐compulsive problems	3.0 (2.8)****, ^††††^	6.4 (3.7)****	0.9 (1.1)	*F*_2,305_ = 123.5	< 0.0001
Affective problems	5.4 (4.6)****	4.4 (3.6)****	0.7 (1.3)	*F*_2,305_ = 66.3	< 0.0001
Anxiety problems	3.4 (2.9)****, ^††^	4.4 (2.9)****	0.6 (1.1)	*F*_2,305_ = 82.3	< 0.0001
Somatic problems	2.0 (2.4)****	2.3 (2.5)****	0.5 (1.0)	*F*_2,305_ = 28.2	< 0.0001
Attention deficit/Hyperactivity problems	8.3 (3.3)****, ^††††^	2.3 (2.4)**	1.3 (1.9)	*F*_2,305_ = 218.9	< 0.0001
Oppositional defiant problems	4.3 (2.9)****	2.4 (2.3)****	1.2 (1.7)	*F*_2,305_ = 50.0	< 0.0001
Conduct problems	4.6 (4.7)****, ^††††^	1.3 (2.2)*	0.6 (1.3)	*F*_2,305_ = 53.7	< 0.0001

*Note:* The Child Behavior Checklist/6–18 was not completed for one patient with ADHD and one patient with OCD at the time of event‐related potential data collection.

Abbreviations: ADHD, attention deficit/hyperactivity disorder; HC, healthy control; OCD, obsessive‐compulsive disorder; SD, standard deviation.

* Compared to HC participants, *p* < 0.05.

** Compared to HC participants, *p* < 0.01.

**** Compared to HC participants, *p* < 0.0001.

^††^ Compared to participants with OCD, *p* < 0.01.

^††††^ Compared to participants with OCD, *p* < 0.0001.

Of the 174 patients, 84 had a lifetime diagnosis of ADHD and 90 a lifetime diagnosis of OCD. Of the 84 patients with ADHD, 29 had a combined presentation, 50 a predominantly inattentive presentation, and 5 a predominantly hyperactive/impulsive presentation. Seven patients with a lifetime diagnosis of ADHD were included in the present study but not in the Liu et al. (2020) study, whereas 15 patients with lifetime diagnoses of both ADHD and OCD were included in the Hanna et al. (2026) study but not in the present study. Patients were excluded if they had a lifetime diagnosis of autism spectrum disorder, anorexia nervosa, schizophrenia, other psychotic disorder, bipolar disorder, or substance‐related disorder. All 136 HC had no history of a specific psychiatric disorder. Lifetime and current psychiatric diagnoses were made independently by two clinicians using all sources of information according to *DSM‐5* criteria (American Psychiatric Association 2013). Participants were excluded if they had a history of intellectual disability, head injury with a loss of consciousness, or chronic neurological disorder other than tics. Because studies have indicated that treatment with a serotonin reuptake inhibitor (SRI) has no effect on the ERN, 55 patients were enrolled taking a stable dose of an SRI but no other non‐stimulant medications (Riesel 2019; Weinberg et al. 2015). Patients taking an SRI were significantly older than patients not taking one (*t*(172) = 2.59, *p* = 0.01, Cohen's *d* = 0.42). The 48 patients with ADHD taking stimulant medications were asked to discontinue them 48 h prior to the EEG.

### 2.2 Diagnostic Instruments

All 310 participants were interviewed with the Schedule for Schizophrenia and Affective Disorders for School‐Aged Children‐Present and Lifetime Version and Schedule for Obsessive‐Compulsive and Other Behavioral Syndromes (Hanna 2013; Kaufman et al. 1997). Parents completed the Child Behavior Checklist 6–18 (CBCL/6–18) about their children (Achenbach and Rescorla 2001; Hudziak et al. 2006).

### 2.3 Stimulus Material and Task Procedures

Participants performed a modified Eriksen flanker task in which arrows appeared on a computer display with congruent (e.g., → → → → →) and incongruent (e.g., → → ← → →) conditions (Eriksen and Eriksen 1974). They were instructed to respond by pressing one of two buttons indicating the direction of the central arrow (i.e., right versus left), while ignoring the adjacent arrows, and to respond as quickly and accurately as possible, placing equal emphasis on speed and accuracy. The flanker task is a test of selective attention and response inhibition that activates the anterior cingulate cortex and pre‐supplementary motor cortex and may provide a more efficient and reliable measure of ERN amplitude than the Stroop or Go/NoGo tasks (Holbrook et al. 2025; Meyer et al. 2013). The stimuli remained on the screen for 250 ms, with an interval of 1500 ms between the response to the onset of the next trial. Each participant was seated 0.65 m directly in front of the computer monitor. Following 40 practice trials, each subject completed 8 blocks of 64 trials with the number of completed trials ranging from 256 to 512. Performance feedback was provided after every block to yield an error rate of approximately 10%, with encouragement to focus on speed if there were fewer than four errors or to focus on accuracy if there were more than 10 errors. Similar feedback has been used in previous studies of the ERN to encourage fast and accurate responses (Meyer et al. 2013). Performance feedback may have helped younger or more inattentive participants stay engaged in the task. However, because the effects of performance feedback were not assessed, its effects on group differences in accuracy and RTV cannot be determined.

### 2.4 Electrophysiological Recording and Data Reduction

The electroencephalogram was recorded from DC‐104 Hz with 64 Ag/AgCl scalp electrodes, two mastoid electrodes, and two vertical and two horizontal electro‐oculogram electrodes, using the BioSemi ActiveTwo system (Amsterdam, the Netherlands). Data were digitized at 512 Hz, referenced to a ground formed from a common mode sense active electrode and driven right leg passive electrode (see http://www.biosemi.com/faq/cms&drl.htm), and rereferenced offline to the average of the two mastoid electrodes. Data were band‐pass filtered 0.1–30 Hz using zero‐phase shift filters. EEG data were screened using automated algorithms that rejected epochs in which absolute voltage exceeded 500 μV and epochs containing peak to peak activity > 500 μV within 200 ms, with a 100 ms moving window, for midline channels (Fz, FCz, Cz, CPz, Pz). Ocular movement artifacts were then corrected using a regression‐based algorithm (Gratton et al. 1983). After ocular correction, individual trials were rejected if they contained absolute amplitudes > 100 μV, a change > 50 μV measured from one data point to the next point, or a maximum voltage difference < 0.5 μV within a trial in any of the midline electrodes.

The mean amplitude of the ERN was computed on error trials in a window from 0 to 80 ms following the incorrect response, relative to a pre‐response baseline of −200 to −50 ms. The mean amplitude of the Pe was computed on error trials in a window from 200 to 400 ms following the erroneous response, compared to a pre‐response baseline of −200 to −50 ms. The CRN and Pc consisted of the same respective measures computed on correct trials. Amplitudes were calculated for electrodes Fz, FCz, Cz, CPz, and Pz, with the focus of the present study on the ERN and CRN measures at Cz and Pe and Pc measures at CPz. Correlational analyses with the ERN and CRN indicate that numerically greater negative values represent higher ERP amplitudes, whereas correlational analyses with the Pe and Pc indicate that numerically greater positive values represent higher ERP amplitudes (Table 4). The ΔERN and ΔPe were calculated by subtracting the CRN from ERN and the Pc from Pe, respectively, as they may isolate neural activity unique to error processing from activity more broadly related to response monitoring (Gehring et al. 2012). A recent study supported the convergent and divergent validity of the ERN, Pe, and ΔPe, but not the ΔERN, across reaction time tasks eliciting response conflict (Clayson et al. 2023).

Behavioral measures included the number of erroneous and correct trials for each subject, as well as accuracy expressed as a percentage of valid trials. Mean reaction times on error and correct trials were calculated separately, and trials were excluded if their reaction times were > 3 standard deviations from the mean. With the 3 standard deviation criterion, the mean percentage of excluded higher reaction time trials was 1.92% in ADHD cases, 1.19% in OCD cases, and 1.22% in HC, suggesting that exclusion may have minimized the estimates of RTV particularly in the ADHD cases. As noted above, the correct trial CVRT was used as the primary index of intrasubject RTV (Elmaghrabi et al. 2020). Reaction time and accuracy after errors were evaluated to determine whether there were group differences in post‐error behavioral adjustments (Gehring et al. 2012). Reaction times were analyzed with group as a between‐subject factor and response type as a within‐subject factor. The mean number of errors per subject contributing to the analysis was 45.0 (SD = 25.2; range = 10–160).

### 2.5 Statistical Analyses

Student *t*‐tests, *χ* ^2^, analysis of variance, and analysis of covariance tests were used to evaluate group differences in demographic, clinical, and behavioral data. Pearson correlation coefficients were used to examine associations of response‐related amplitudes with age, behavioral measures, and clinical measures. Six electrocortical indicators (ERN, CRN, Pe, Pc, ΔERN, ΔPe) of performance monitoring were analyzed separately using a repeated‐measure analysis of covariance with group (ADHD, OCD, HC) as a between‐subject factor, response type (correct, incorrect) as a within‐subject factor, and age as a covariate (Gehring et al. 2012). Primary analyses were done with the ERN, CRN, Pe, and Pc; secondary analyses were done with the ΔERN and ΔPe to allow comparisons with previous ERP studies of ADHD and OCD. Similar analyses were done to compare brain potentials in male and female participants and medicated and unmedicated OCD cases. Cohen's effect size conventions were used to describe the magnitude of effects (small: *d* ≥ 0.20; medium: *d* ≥ 0.50; large: *d* ≥ 0.80) (Cohen 1992).

A multiple linear regression analysis was done with all participants to examine the association of correct trial CVRT with age, psychiatric diagnosis using two diagnosis dummy variables, and ERN, CRN, Pe, and Pc amplitudes. A similar multiple linear regression analysis was completed with all participants to examine the association of correct trial CVRT with age, the four brain potentials, and CBCL/6–18 *DSM*‐Oriented Scale scores (Affective, Anxiety, Somatic, AD/H, Oppositional Defiant, and Conduct Problems) to assess the specificity of the relationship between CVRT and AD/H Problems scores (Achenbach and Rescorla 2001). The CBCL/6–18 Obsessive Compulsive Problems Scale was not included in this analysis because it shares items with the original *DSM*‐Oriented Scales (Hudziak et al. 2006). In addition to the two primary full models, reduced models were derived using backward stepwise regression analysis for confirmatory analyses. No interaction terms were included in either model. Analyses were performed with JMP 18 software. All tests were two‐tailed with *α* = 0.05.

## 3 Results

### 3.1 Clinical Data in Patients With ADHD, Patients With OCD, and Healthy Controls

CBCL/6–18 *DSM*‐Oriented Scale scores were significantly elevated in ADHD and OCD cases compared to HC as expected (Table 1). Moreover, AD/H Problems scores were significantly higher in ADHD cases compared to OCD cases (*t*(170) = 13.9, *p* < 0.0001, Cohen's *d* = 2.11), whereas OC Problems scores were significantly higher in OCD cases relative to ADHD cases (*t*(170) = 6.88, *p* < 0.0001, Cohen's *d* = 1.05).

### 3.2 Behavioral Data in Patients With ADHD, Patients With OCD, and Healthy Controls

Participants were significantly more accurate on congruent than incongruent trials of the flanker task (paired *t*(309) = 31.9, *p* < 0.0001). Age had significant positive correlations with overall accuracy (*r* = 0.21, *p =* 0.0002), post‐correct accuracy (*r* = 0.17, *p* = 0.002), and post‐error accuracy (*r* = 0.29, *p* < 0.0001). There were no significant sex differences in error number or overall accuracy (both *p* values > 0.10). OCD cases made significantly more errors than ADHD cases (*p* < 0.05, Cohen's *d* = 0.37) and HC (*p* < 0.05, Cohen's *d* = 0.33). OCD cases had significantly lower overall accuracy than HC (*p* = 0.007, Cohen's *d* = 0.34) but not ADHD cases (*p* > 0.05) (Table 2).

**TABLE 2:** Behavioral data in participants with ADHD, participants with OCD, and HC participants.

Variable	ADHD	OCD	HC	ADHD vs. OCD vs. HC	
	(*n* = 84)	(*n* = 90)	(*n* = 136)	Participants	*p*
	Mean (SD)	Mean (SD)	Mean (SD)	Test statistic	
Total number of trials	456.5 (72.4)	475.9 (58.6)	469.3 (70.9)	*F*_2,306_ = 1.52	0.22
Total number of error trials	41.4 (23.7)^†^	51.4 (29.6)*	42.9 (22.3)	*F*_2,306_ = 4.37	0.01
Accuracy on all trials	0.90 (0.06)	0.88 (0.07)**	0.90 (0.05)	*F*_2,306_ = 3.89	0.02
Accuracy on congruent trials	0.96 (0.04)	0.96 (0.05)	0.97 (0.03)	*F*_2,306_ = 1.80	0.17
Accuracy on incongruent trials	0.83 (0.09)^†^	0.80 (0.10)**	0.84 (0.08)	*F*_2,306_ = 4.12	0.02
Accuracy after correct trials	0.90 (0.06)	0.88 (0.07)**	0.90 (0.05)	*F*_2,306_ = 3.75	0.02
Accuracy after incorrect trials	0.89 (0.09)	0.88 (0.10)*	0.90 (0.08)	*F*_2,306_ = 2.72	0.04
Error reaction time (ms)	568.6 (283.1)***, ^††^	446.3 (210.1)	457.7 (180.8)	*F*_2,306_ = 9.53	< 0.0001
Error reaction time (SD)	248.4 (191.9)****, ^††††^	141.4 (162.5)	155.6 (146.0)	*F*_2,306_ = 12.83	< 0.0001
Error trial CVRT	0.40 (0.20)***, ^††††^	0.27 (0.17)	0.30 (0.19)	*F*_2,306_ = 11.16	< 0.0001
Correct reaction time (ms)	580.9 (181.7)****, ^††††^	488.4 (147.6)	508.1 (146.5)	*F*_2,306_ = 10.94	< 0.0001
Correct reaction time (SD)	176.0 (106.5)****, ^††††^	116.3 (92.8)	127.4 (87.5)	*F*_2,306_ = 12.63	< 0.0001
Correct trial CVRT	0.28 (0.09)****, ^††††^	0.22 (0.09)	0.23 (0.09)	*F*_2,306_ = 14.01	< 0.0001
Reaction time after error trials (ms)	577.9 (184.3)****, ^††††^	482.5 (147.0)	502.1 (144.0)	*F*_2,306_ = 11.08	< 0.0001
Reaction time after correct trials (ms)	577.4 (187.6)****, ^††††^	481.4 (149.7)	501.4 (147.7)	*F*_2,306_ = 11.25	< 0.0001
Post‐error slowing (ms)	11.4 (126.4)*	40.4 (78.4)	46.7 (65.0)	*F*_2,306_ = 4.12	0.02

*Note:* Analysis of covariance tests were used with age as a covariate to evaluate group differences in behavioral data.

Abbreviations: ADHD, attention deficit/hyperactivity disorder; CVRT, coefficient of variation of reaction time; HC, healthy control; ms, milliseconds; OCD, obsessive‐compulsive disorder; RT, reaction time; SD, standard deviation.

* Compared to HC participants, *p* < 0.05.

** Compared to HC participants, *p* < 0.01.

*** Compared to HC participants, *p* < 0.001.

**** Compared to HC participants, *p* < 0.0001.

^†^ Compared to participants with OCD, *p* < 0.05.

^††^ Compared to participants with OCD, *p* < 0.01.

^††††^ Compared to participants with OCD, *p* < 0.0001.

Correct responses were significantly slower than incorrect responses (paired *t*(309) = 6.61, *p* < 0.0001). Age had significant negative correlations with reaction time on correct (*r* = −0.60, *p* < 0.0001) and incorrect trials (*r* = −0.49, *p* < 0.0001) and a significant positive correlation with post‐error slowing (*r* = 0.21, *p* = 0.0002). There were no significant sex differences in reaction time on correct or incorrect trials or post‐error slowing (all *p* values > 0.30). In a repeated‐measures ANOVA examining flanker task CVRT, with response type (correct, incorrect) as a within‐subject factor and group (ADHD, OCD, HC) as a between‐subject factor, there was a significant main effect for group (Exact *F*_2,306_ = 14.13, *p* < 0.0001) and a significant interaction between group and CVRT response type (Exact *F*_2,306_ = 4.32, *p* = 0.014). Table 4 provides a correlation matrix for age, flanker task accuracy, error and correct trial CVRT, and brain potential measures.

Correct trial CVRT was significantly increased in ADHD compared to OCD cases (*F*_1,171_ = 25.60, *p* < 0.0001, Cohen's *d* = 0.71) and HC (*F*_1,217_ = 18.20, *p* < 0.0001, Cohen's *d* = 0.53). Error trial CVRT was also significantly higher in ADHD cases compared to OCD cases (*F*_1,171_ = 19.78, *p* < 0.0001, Cohen's *d* = 0.67) and HC (*F*_1,217_ = 14.12, *p* < 0.001, Cohen's *d* = 0.50). Similar significant increases in correct and error trial reaction time and correct and error trial reaction time standard deviation were found in ADHD compared to OCD cases and HC (Table 2). Correct trial CVRT had a significant positive correlation with AD/H Problems Scale scores in ADHD (*r* = 0.40, *p* = 0.0002) but not OCD cases (*p* = 0.54) or HC (*p* = 0.45). Error trial CVRT had a significant positive correlation with AD/H Problems Scale scores in ADHD (*r* = 0.40, *p* = 0.0002) but not OCD cases (*p* = 0.41) or HC (*p* = 0.36).

### 3.3 Event‐Related Potential Data in ADHD Cases, OCD Cases, and Healthy Controls

ERN amplitude was significantly increased (more negative) compared to CRN amplitude (paired *t* (309) = −9.79, *p* < 0.0001). Pe amplitude was significantly increased (more positive) compared to Pc amplitude (paired *t* (309) = 28.56, *p* < 0.0001). Age in all participants had significant correlations with the ERN (*r* = −0.24, *p* < 0.0001), CRN (*r* = 0.24, *p* < 0.0001), ΔERN (*r* = −0.41, *p* < 0.0001), and Pc (*r* = 0.17, *p* = 0.003) but not Pe or ΔPe (both *p* values > 0.05) (Table 4).

**FIGURE 1:**
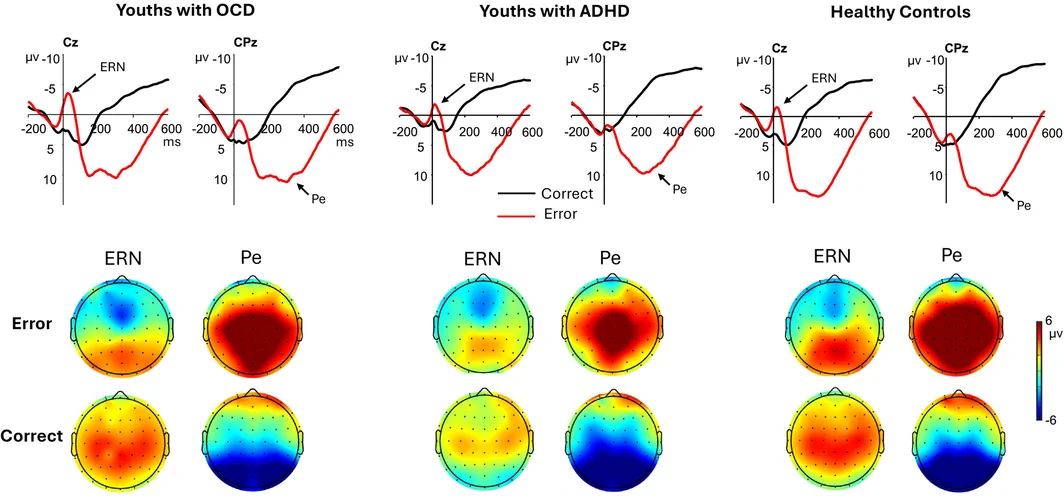
Grand averages of electroencephalogram (EEG) recordings in 84 patients with attention‐deficit/hyperactivity disorder, 90 patients with obsessive‐compulsive disorder (OCD), and 136 healthy controls (HC). The top images depict response‐locked grand average waveforms recorded at the Cz and CPz electrodes for correct and incorrect responses. Responses occurred at 0 ms. The mean amplitude of the error‐related negativity (ERN) was computed in a window 0 to 80 ms after incorrect response trials. The mean amplitude of the correct response negativity (CRN) consisted of the same measure computed on correct response trials. The ΔERN was calculated by subtracting the CRN from the ERN. The mean amplitude of the error positivity (Pe) was computed in a window 200 to 400 ms after incorrect response trials. The mean amplitude of the correct positivity (Pc) consisted of the same measure computed on correct response trials. The ΔPe was calculated by subtracting the Pc from the Pe. The positivity visible at about 200 ms before time zero reflects pre‐response rather than pre‐stimulus activity. Because the next trial did not begin until 1500 ms after the response, this positivity is unlikely to reflect anticipation of the subsequent stimulus. It may instead reflect response‐preparatory or other slow activity associated with the current trial. The bottom images depict the topography of mean amplitudes of erroneous and correct waveforms measured between 0 and 80 ms and between 200 and 400 ms.

CRN amplitude was significantly increased in ADHD cases compared to HC (*F*_1,217_ = 8.88, *p* = 0.003, Cohen's *d* = 0.41), with a significant age effect (*F*_1,217_ = 12.66, *p* = 0.0005) (Table 3; Figure 1). ERN amplitude was significantly higher in OCD cases compared to HC (*F*_1,223_ = 8.08, *p* = 0.005, Cohen's *d* = 0.40), with a significant effect for age (*F*_1,223_ = 7.05, *p* = 0.008). The ΔERN was significantly raised in OCD compared to ADHD cases (*F*_1,171_ = 11.73, *p* = 0.0008, Cohen's *d* = 0.52), with a significant effect for age (*F*_1,171_ = 42.35, *p* < 0.0001). There were trends for differences between ADHD and OCD cases in ERN (*F*_1,171_ = 3.71, *p* = 0.06, Cohen's *d* = 0.32) and CRN amplitudes (*F*_1,171_ = 2.95, *p* = 0.09, Cohen's *d* = 0.28).

**TABLE 3:** Event‐related electrocortical potential data in participants with ADHD, participants with OCD, and HC participants.

Variable	ADHD	OCD	HC	ADHD vs. OCD vs. HC	
	(*n* = 84)	(*n* = 90)	(*n* = 136)	Participants	*p*
	Mean (SD)	Mean (SD)	Mean (SD)	Test statistic	
Error‐related negativity, FCz (μV)	−2.65 (4.81)	−4.25 (5.99)	−2.67 (5.72)	*F*_2,306_ = 2.29	0.10
Correct response negativity, FCz (μV)	1.00 (4.88)**, ^†^	2.59 (4.68)	2.90 (4.02)	*F*_2,306_ = 4.89	0.008
ΔERN, FCz (μV)	−3.65 (5.87)*, ^†††^	−6.85 (6.32)	−5.57 (6.57)	*F*_2,306_ = 5.37	0.005
Error‐related negativity, Cz (μV)	−0.08 (4.81)	−1.84 (6.13)**	0.59 (6.12)	*F*_2,306_ = 4.57	0.01
Correct response negativity, Cz (μV)	1.98 (5.21)**	3.44 (5.10)	4.03 (4.82)	*F*_2,306_ = 4.40	0.01
ΔERN, Cz (μV)	−2.06 (5.87)^†††^	−5.28 (6.38)	−3.44 (6.66)	*F*_2,306_ = 5.37	0.005
Error positivity, CPz (μV)	8.56 (9.00)**	10.32 (8.42)	12.64 (9.69)	*F*_2,306_ = 5.34	0.005
Correct positivity, CPz (μV)	−5.57 (6.49)	−3.40 (8.04)	−5.38 (7.18)	*F*_2,306_ = 2.31	0.10
ΔPe, CPz (μV)	14.14 (9.77)**	13.72 (9.42)**	18.02 (9.39)	*F*_2,306_ = 7.07	0.001

*Note:* Analysis of covariance tests were used with age as a covariate to evaluate group differences in event‐related electrocortical potential data.

Abbreviations: ΔERN, error‐related negativity minus correct‐response negativity; ΔPe, error positivity minus correct positivity; ADHD, attention deficit/hyperactivity disorder; CRN, correct‐response negativity; ERN, error‐related negativity; HC, healthy control; OCD, obsessive‐compulsive disorder; Pc, correct positivity; Pe, error positivity; SD, standard deviation.

* Compared to HC participants, *p* < 0.05.

** Compared to HC participants, *p* < 0.01.

^†^ Compared to participants with OCD, *p* < 0.05.

^†††^ Compared to participants with OCD, *p* < 0.001.

**TABLE 4:** Correlation matrix for correct trial CVRT, error trial CVRT, age, flanker task accuracy, error‐related negativity (ERN), correct‐response negativity (CRN), and ERN minus CRN at electrode Cz, and error positivity (Pe), correct positivity (Pc), and Pe minus Pc at electrode CPz in ADHD cases, OCD cases, and healthy controls.

	Correct trial CVRT	Error trial CVRT	Age	Flanker task accuracy	ERN Cz (μV)	CRN Cz (μV)	Pe CPz (μV)	Pc CPz (μV)
Correct trial CVRT	—	0.78****	−0.51****	−0.28****	0.04	−0.34****	−0.25****	−0.05
Error trial CVRT	0.78****	—	−0.39****	−0.08	0.08	−0.36****	−0.22****	−0.09
Age	−0.51****	0.39****	—	0.21***	−0.24****	0.24****	0.03	0.17**
Flanker task accuracy	−0.28****	−0.08	0.21***	—	−0.13*	−0.10	0.18**	−0.10
ERN, Cz (μV)	0.04	0.08	−0.24****	−0.13*	—	0.31****	0.26****	−0.14*
CRN, Cz (μV)	−0.34****	−0.36****	0.24****	−0.10	0.31****	—	0.41****	0.37****
ΔERN, Cz (μV)	0.30****	0.35****	−0.41****	0.04	0.67****	−0.50****	−0.09	−0.41****
Pe, CPz (μV)	−0.25****	−0.22****	0.03	0.18**	0.26***	0.41****	—	0.34****
Pc, CPz (μV)	−0.05	−0.09	0.17**	−0.10	−0.14*	0.37****	0.34****	—
ΔPe, CPz (μV)	−0.20***	−0.14*	−0.09	0.25****	0.35***	0.11*	0.74****	−0.43****

Abbreviations: ADHD, attention deficit/hyperactivity disorder; CRN, correct response negativity; CVRT, coefficient of variation of reaction time; ERN, error‐related negativity; ERN, error‐related negativity; OCD, obsessive‐compulsive disorder; Pc, correct positivity; Pe, error positivity.

* *p* < 0.05.

** *p* < 0.01.

*** *p* < 0.001.

**** *p* < 0.0001.

**TABLE 5:** Multiple linear regression model for flanker task correct trial CVRT as dependent variable and age, psychiatric diagnosis, error‐related negativity and correct response negativity at electrode Cz, and error positivity and correct positivity at electrode CPz as predictors in ADHD cases, OCD cases, and healthy controls.

Full model	Regression						Correlation	
	*R* ^2^	*β*	*β* (SE)	*t*	*p*	*F*	*r* (bivariate)	*r* (partial)
	0.407				< 0.0001	29.65		
Age		−0.02	0.002	−9.59	< 0.0001		−0.51	−0.48
Pe, CPz (μV)		−0.002	0.0005	−4.05	< 0.0001		−0.25	−0.23
Pc, CPz (μV)		0.003	0.0007	3.83	0.0002		−0.05	0.22
CRN, Cz (μV)		−0.004	0.001	−3.58	0.0004		−0.34	−0.20
ADHD		0.03	0.01	3.04	0.003		0.26	0.17
OCD		−0.02	0.01	−2.17	0.03		−0.17	−0.12
ERN, Cz (μV)		0.001	0.0009	0.90	0.37		0.04	0.05

Reduced model*	Regression						Correlation	
	*R* ^2^	*β*	*β* (SE)	*t*	*p*	*F*	*r* (bivariate)	*r* (partial)
	0.406				< 0.0001	34.48		
Age		−0.02	0.002	−10.43	< 0.0001		−0.51	−0.51
Pe, CPz (μV)		−0.002	0.0005	−3.95	< 0.0001		−0.25	0.22
Pc, CPz (μV)		0.002	0.0006	3.73	0.0002		−0.05	−0.21
CRN, Cz (μV)		−0.003	0.001	−3.51	0.0005		−0.34	−0.20
ADHD		0.03	0.01	3.10	0.002		0.26	0.18
OCD		−0.02	0.01	−2.28	0.02		−0.17	−0.13

Abbreviations: ADHD, attention deficit/hyperactivity disorder; CRN, correct response negativity; CVRT, coefficient of variation of reaction time; ERN, error‐related negativity; OCD, obsessive‐compulsive disorder; Pc, correct positivity; Pe, error positivity; SE, standard error.

* After backward stepwise deletion of nonsignificant variable.

**TABLE 6:** Multiple linear regression model for flanker task correct trial CVRT as dependent variable and age, Child Behavior Checklist/6–18 *DSM*‐Oriented Scales, error‐related negativity and correct response negativity at electrode Cz, and error positivity and correct positivity at electrode CPz as predictors in ADHD cases, OCD cases, and healthy controls.

Full model	Regression						Correlation	
	*R* ^2^	*β*	*β* (SE)	*t*	*p*	*F*	*r* (bivariate)	*r* (partial)
	0.393				< 0.0001	17.44		
Age		−0.01	0.002	−8.57	< 0.0001		−0.51	−0.45
Pe, CPz (μV)		−0.002	0.0005	−4.01	< 0.0001		−0.25	−0.22
CRN, Cz (μV)		−0.004	0.001	3.52	0.0005		−0.34	−0.20
Pc, CPz (μV)		0.002	0.001	3.50	0.0005		−0.05	0.20
CBCL AD/H problems		0.005	0.002	3.17	0.002		0.31	0.18
CBCL anxiety problems		−0.003	0.002	−1.23	0.22		0.09	−0.07
ERN, Cz (μV)		0.001	0.001	1.02	0.31		0.04	0.06
CBCL oppositional defiant problems		−0.002	0.003	−0.76	0.45		0.18	−0.04
CBCL Affective Problems		0.001	0.002	0.51	0.61		0.08	0.03
CBCL somatic problems		−0.001	0.003	−0.24	0.81		0.03	−0.01
CBCL conduct problems		0.0003	0.002	0.13	0.89		0.03	−0.01

Reduced model*	Regression						Correlation	
	*R* ^2^	*β*	*β* (SE)	*t*	*p*	*F*	*r* (bivariate)	*r* (partial)
	0.384				< 0.0001	37.63		
Age		−0.015	0.002	−9.51	< 0.0001		−0.51	−0.48
Pe, CPz (μV)		−0.002	0.0005	−3.80	0.0002		−0.25	−0.21
CBCL AD/H problems		0.004	0.001	3.68	0.0003		0.31	0.21
CRN, Cz (μV)		−0.003	0.001	−3.31	0.001		−0.34	−0.19
Pc, CPz (μV)		0.002	0.0007	3.29	0.001		−0.05	0.19

*Note:* The Child Behavior Checklist/6–18 was not completed for one patient with ADHD and one patient with OCD at the time of event‐related potential data collection.

Abbreviations: AD/H, attention‐deficit/hyperactivity; ADHD, attention‐deficit/hyperactivity disorder; CBCL, Child Behavior Checklist; CRN, correct response negativity; CVRT, coefficient of variation of reaction time; ERN, error‐related negativity; OCD, obsessive‐compulsive disorder; Pc, correct positivity; Pe, error positivity; SE, standard error.

* After backward stepwise deletion of nonsignificant variables.

Pe amplitude at CPz was significantly decreased in ADHD cases compared to HC (*F*_1,217_ = 9.60, *p* = 0.002, Cohen's *d* = 0.43), without a significant age effect (*F*_1,217_ = 0.10, *p* = 0.76) (Table 3; Figure 1). The ΔPe was also significantly decreased in ADHD cases compared to HC (*F*_1,217_ = 8.89, *p* = 0.003, Cohen's *d* = 0.41), with a trend for an age effect (*F*_1,217_ = 3.44, *p* = 0.06), and OCD cases compared to HC (*F*_1,223_ = 11.08, *p* = 0.001, Cohen's *d* = 0.46), without a significant age effect (*F*_1,223_ = 0.46, *p* = 0.50).

There were no significant sex differences in any brain potentials (all *p* values > 0.5). There were no significant differences in any brain potentials between medicated and unmedicated OCD cases (all *p* values > 0.15).

### 3.4 Flanker Task Correct Trial CVRT, Age, Diagnostic Measures, and Event‐Related Potential Data in Patients With ADHD, Patients With OCD, and Healthy Controls

In a multiple linear regression analysis examining the association of correct trial CVRT with age, psychiatric diagnosis, and ERN, CRN, Pe, and Pc amplitudes, CVRT had significant associations with age, ADHD, OCD, Pe, Pc, and CRN in the full model. A backward stepwise regression analysis indicated that only those variables were significantly associated with CVRT in the reduced model (Table 5). Higher CVRT was associated with ADHD as well as lower (less positive) Pe, higher (more positive) Pc, and higher (more negative) CRN amplitudes.

In a similar multiple linear regression analysis examining the associations of correct trial CVRT with age, the four brain potentials, and CBCL/6–18 *DSM*‐Oriented Scale scores, CVRT had significant associations with age, AD/H Problems scores, Pe, Pc, and CRN in the full model. A backward stepwise regression analysis indicated that only those variables were significantly associated with CVRT in the reduced model (Table 6). Higher CVRT was associated with higher AD/H Problems scores as well as lower Pe, higher Pc, and higher CRN amplitudes.

## 4 Discussion

This study was done with a large sample of older children and adolescents with a lifetime diagnosis of ADHD, OCD, or no psychiatric disorder using a flanker task to compare the three groups in their CVRT, accuracy, and neural indices of performance monitoring. As the performance monitoring neural system may be associated with RTV, multiple linear regression analyses were used to examine the association of correct trial CVRT with age, psychiatric diagnostic measures, and the four brain potentials involved in error monitoring.

### 4.1 Reaction Time Variability and Accuracy in ADHD and OCD

Correct and error trial CVRT were increased in ADHD cases compared to OCD cases and HC. Cohen's *d* values for these comparisons ranged from 0.50 to 0.71, indicating a medium effect size, which is comparable to the Hedges' *g* of 0.76 reported in a meta‐analysis of ADHD studies in children and adolescents (Kofler et al. 2013). However, as noted above, the exclusion of trials with reaction times > 3 standard deviations from the mean may have resulted in a greater underestimate of the CVRT in the ADHD group than in the other two groups. A previous comparison of RTV in ADHD and OCD cases has not been reported to our knowledge. Increased RTV has been noted in youths with bipolar disorder (Brotman et al. 2009), adolescents with psychotic‐like experiences (Wallace and Linscott 2018), and youths at high risk for bipolar disorder and schizophrenia (Johnsen et al. 2024). Thus, increased RTV is a key neurocognitive marker of several forms of severe childhood psychopathology. In contrast to the RTV findings in ADHD, OCD cases made more errors than HC and ADHD cases and had lower overall accuracy than HC.

### 4.2 Electrocortical Markers of Performance Monitoring in ADHD and OCD

CRN amplitude was increased in ADHD cases compared to HC, whereas the ΔERN was decreased in ADHD compared to OCD cases. Our finding of an enlarged CRN in ADHD requires replication in other large pediatric ADHD samples and is consistent with the hypothesis that ADHD is associated with a delayed maturation of the CRN (Boen et al. 2022; Liu et al. 2020; Overbye et al. 2019; Tamnes et al. 2013). Both Pe amplitude and the ΔPe were decreased in ADHD cases relative to HC. Our Pe results are consistent with a meta‐analysis that concluded a reduced Pe is associated more reliably with ADHD than a reduced ERN (Kaiser et al. 2020) and with the recent finding of a blunted Pe in a large study of adolescents with ADHD (Figuracion et al. 2024). The Pe and CRN results taken together suggest ADHD may be characterized by an imbalance in error‐related and correct‐related brain activity.

ERN amplitude was increased in OCD cases compared to HC, which is consistent with numerous other reports (Bellato et al. 2021; Lamothe et al. 2025; Michael et al. 2021; Riesel 2019). However, the OCD cases were less accurate than the HC on the flanker task in our study, which is contrary to studies of adults with OCD (Bellato et al. 2021; Michael et al. 2021; Riesel 2019). From the perspective of Attentional Control Theory, it is possible that persistent obsessions and severe anxiety in pediatric OCD interfere with the efficient functioning of the goal‐directed attentional system that may not be as well compensated for by an enhanced ERN in youths as in adults (Eysenck et al. 2007; Moser et al. 2013). A similar model has proposed that an overflow of obsessions causes an overload on the executive system, which consumes cognitive resources and results in neurocognitive impairments (Abramovitch et al. 2012).

The ΔPe was decreased in OCD cases relative to HC, which requires replication in other large pediatric OCD samples. Studies examining the ERN and ΔPe in other psychiatric disorders may determine whether the combination of an increased ERN and decreased ΔPe is specific to pediatric OCD. Consistent with the significant correlations of the Pe and ΔPe with flanker task accuracy, the reduced Pe and ΔPe in ADHD cases and reduced ΔPe in OCD cases may reflect defects in the post‐decisional evidence accumulation process that impair decision accuracy, decision confidence, and subsequent behavioral adjustments (Boldt and Yeung 2015; Desender et al. 2021; Murphy et al. 2012; Steinhauser and Yeung 2010). Previous studies with adults have found that ADHD and OCD share similar neuropsychological impairments, especially in executive functions, which suggests those impairments may be associated with a decreased ΔPe (Abramovitch et al. 2012). Future treatment studies may determine whether interventions increasing the ΔPe improve either condition.

### 4.3 RTV and Neural Indices of Performance Monitoring in ADHD and OCD

The association of correct trial CVRT with age, diagnostic measures, and neural correlates of error monitoring was examined in two multiple regression analyses. In the first full model, CVRT was associated with age, ADHD, OCD, and CRN, Pe, and Pc amplitudes. In the second full model, correct trial CVRT was associated with age, AD/H Problems scores, and CRN, Pe, and Pc amplitudes. The association between CVRT and AD/H Problems scores but not the other five CBCL/6–18 *DSM*‐Oriented Scale scores is consistent with a recent report that attentiveness modulates RTV in children and adolescents (Aristodemou et al. 2024). Slower reaction times have been associated with higher CRN amplitudes in other studies (Files et al. 2021; Luu et al. 2000), which raises the possibility that an augmented CRN in ADHD may reflect the delayed responses or attentional lapses associated with the disorder. The observation of an association between RTV and neural indices of error monitoring in older children and adolescents has not been reported previously to our knowledge and requires further examination in pediatric populations, given our limited understanding of the electrophysiology of higher RTV in ADHD and other severe forms of childhood psychopathology (Brotman et al. 2009; Wallace and Linscott 2018; Tamm et al. 2025). Since ADHD is associated with increased RTV and a possible imbalance in error‐related and correct‐related brain activity, future studies may determine whether the error monitoring neural system mediates the relationship between ADHD polygenic risk scores and RTV (Moses et al. 2022).

### 4.4 Neurobiological Correlates of ADHD, OCD, and Reaction Time Variability

A comparative meta‐analysis found that gray matter volume (GMV) and activity were decreased in the basal ganglia and insula in ADHD cases compared to OCD cases and controls and that both measures were increased in OCD cases relative to controls (Norman et al. 2016). Furthermore, activity was reduced in the ADHD cases predominantly in the right ventrolateral prefrontal cortex (VLPFC), whereas GMV and activity were reduced in the OCD cases in the rostral and dorsal anterior cingulate and medial prefrontal cortex. Ventromedial prefrontal GMV was reduced in both disorders relative to controls. The findings support the hypotheses of a developmental delay in VLPFC‐insular‐striatal networks in ADHD (Rubia et al. 2014; Shaw et al. 2014) and an alteration in frontostriatal development in OCD with larger and overactive basal ganglia that are poorly controlled by underdeveloped and underactive medial frontal structures of top‐down control (Gillan and Robbins 2014; Melloni et al. 2012). Studies of fractional anisotropy using diffusion tensor imaging in adults with ADHD have found that increased RTV is associated with reduced microstructural integrity of the superior longitudinal fasciculus (Wolfers et al. 2015) and altered white matter structural properties of the corticothalamic tract (Kölle et al. 2022).

Reductions in GMV and activity in the anterior cingulate and medial prefrontal cortex do not preclude increased error‐related brain activity in those regions during tasks that elicit response conflict in patients with OCD (Bellato et al. 2021; Hanna et al. 2026; Lamothe et al. 2025; Michael et al. 2021; Riesel 2019). The paradox between reduced GMV or resting activity in parts of the prefrontal‐cingulate network and an enlarged ERN amplitude in OCD arises from structural loss compared to functional overactivity during specific cognitive tasks. More recent work has delineated the functional imbalance in the ventral and dorsal frontostriatal circuits in OCD using computational modeling (Naze et al. 2025) and mapped functional striatal gradients in OCD that provide a spatial representation of continuous changes in whole‐brain connectivity within striatal regions (Webb et al. 2025).

### 4.5 Limitations of the Study

Our study has limitations requiring further consideration. Participants were primarily Caucasian and treatment was uncontrolled; however, it is doubtful that ERP amplitudes would be different in a more diverse or untreated sample (Bellato et al. 2021; Boen et al. 2022; Riesel 2019; Tamnes et al. 2013). Age and sex matching of the HC were less exact than in our previous studies of ADHD and OCD because the ADHD group had a male majority and the OCD group a female majority (Hanna et al. 2026; Liu et al. 2020). No teacher ratings of ADHD behaviors were obtained. Youths with lifetime diagnoses of both ADHD and OCD were excluded from this study; however, a recent study found lower accuracy and higher RTV on the Attention Network Test in children with ADHD and children with ADHD and comorbid anxiety disorder compared to HC (Boen et al. 2026). Youths with a history of bipolar disorder or psychotic symptoms were excluded, but some participants may have had an increased risk for bipolar or psychotic disorders (Brotman et al. 2009; Wallace and Linscott 2018; Johnsen et al. 2024). No corrections were made for multiple statistical comparisons, although the main ERP findings were present in our two previous studies of ADHD and OCD (Hanna et al. 2026; Liu et al. 2020). Many of our findings are correlational, requiring experimental studies to establish any causal relationships between the variables. The study examined inter‐individual measures of RTV, whereas intra‐individual analyses are necessary to isolate within‐subject trial‐to‐trial variance (LoTemplio et al. 2026; Tamm et al. 2025). Furthermore, an Ex‐Gaussian decomposition may provide a more informative analysis of attentional lapses than the CVRT (Bella‐Fernández et al. 2024; Tamm et al. 2012, 2025).

## 5 Conclusions

ADHD and OCD are common psychiatric disorders that may be differentiated by neurocognitive measures and neural indices of error monitoring (Figuracion et al. 2024; Hanna et al. 2026; Kaiser et al. 2020; Kofler et al. 2013; Lamothe et al. 2025; Liu et al. 2020; Lutz et al. 2021; Tamm et al. 2012). The study provides further evidence that ADHD is associated with increased RTV, increased CRN amplitude, and decreased Pe amplitude and ΔPe, whereas OCD is characterized by decreased flanker task accuracy, increased ERN amplitude, and decreased ΔPe. Multiple linear regression analyses found that RTV had significant associations with age, diagnostic measures, and CRN, Pe, and Pc amplitudes. Further research on RTV and neural indices of performance monitoring using Ex‐Gaussian decomposition and other computational methods may lead to more detailed and testable mechanistic models of ADHD and OCD (Killeen et al. 2013; Naze et al. 2025; Pine and Leibenluft 2015; Tamm et al. 2025; Ziegler et al. 2016).

## Author Contributions

**Paul D. Arnold:** conceptualization, investigation, funding acquisition, methodology, validation, writing – review and editing. **Barbara S. Hanna:** investigation, methodology, validation, writing – review and editing, data curation. **Yanni Liu:** conceptualization, investigation, methodology, validation, writing – review and editing, data curation. **Gregory L. Hanna:** conceptualization, investigation, funding acquisition, writing – original draft, methodology, validation, writing – review and editing, data curation, project administration. **William J. Gehring:** conceptualization, investigation, funding acquisition, writing – review and editing, validation, methodology, project administration.

## Funding

This study was funded by the National Institute of Mental Health of the National Institutes of Health (grant R01MH101493).

## Ethics Statement

All tasks and procedures were approved by the University of Michigan Medical School Institutional Review Board. Participants or their parents gave written informed consent in accordance with the Declaration of Helsinki.

## Conflicts of Interest

The authors declare no conflicts of interest.

## Data Availability

Data and materials are available upon request to the corresponding author.
